# Access site complications following transfemoral coronary procedures: comparison between traditional compression and angioseal vascular closure devices for haemostasis

**DOI:** 10.1186/s12872-015-0022-4

**Published:** 2015-05-09

**Authors:** Pei-Jung Wu, Yu-Tzu Dai, Hsien-Li Kao, Chin-Hao Chang, Meei-Fang Lou

**Affiliations:** Department of Nursing, Taipei Veterans General Hospital, 201, Sec. 2, Shipai Road, Taipei City, 11217 Taiwan; School of Nursing, College of Medicine, National Taiwan University, 1, Sec. 1, Jen-Ai Road, Taipei City, 10063 Taiwan; Department of Internal Medicine, College of Medicine, National Taiwan University, 1, Sec. 1, Jen-Ai Road, Taipei City, 10063 Taiwan; Department of Internal Medicine, Division of Cardiology, National Taiwan University Hospital, 7, Chung Shan S. Rd., Taipei City, 10002 Taiwan; Department of Medicine Research, National Taiwan University Hospital, 7, Chung Shan S. Rd., Taipei City, 10002 Taiwan

**Keywords:** Coronary procedures, Manual compression, Vascular closure device, Angioseal, Observation

## Abstract

**Background:**

Vascular closure devices such as angioseal are used as alternatives to traditional compression haemostasis. Although the safety and efficacy of angioseal are confirmed, their use remains controversial because of the potential complications of these devices compared with those of traditional compression haemostasis. The aim of this study was to compare the access site complication rate, the predictive factors for these complications, and patient comfort levels after coronary procedures with traditional compression or angioseal haemostasis.

**Methods:**

Data were collected from a cardiac unit in a medical center in northern Taiwan. A total of 130 adult patients were recruited and equally divided into two groups according to the method of haemostasis used after the coronary procedure: a traditional compression group and an angioseal group. We observed the incidence of access site complications, including bleeding, oozing, haematoma formation, and arteriovenous fistula formation. In addition, we used a 0–10 numeric rating scale to assess soreness, numbness, and back and groin access site pain after 1 h of catheter removal and immediately before getting out of bed.

**Results:**

The overall incidence of complications was 3.8 % (*n* = 5), which was not significantly different between the two groups (*p* = .06). The propensity score—adjusted multivariate analyses revealed that the only independent predictor for access site complications was an age of >70 years (OR, 10.44; 95 % CI, 1.81–60.06; *p* = .009). Comfort levels were higher in the angioseal group than in the traditional compression group.

**Conclusions:**

Angioseal used after coronary procedures did not increase the incidence of complications relative to that associated with traditional compression haemostasis; however, it increased patient comfort levels. Health personnel should pay special attention to the predictive factor for access site complications after coronary procedures, such as age >70 years.

## Background

Coronary angiography (CA) and percutaneous coronary intervention (PCI) are common methods for confirming the severity of coronary artery occlusion and treating (e.g., stent placement) coronary artery disease. To prevent access site bleeding, patients need to lie in bed after this procedure. The duration of bed confinement after coronary procedures differs according to unit protocol. Because of the use of compression bandages on puncture sites and long durations of bed confinement, hip and leg mobility become restricted, causing common complaints such as back soreness and related problems. Patients who use a compression bandage for haemostasis after coronary procedures are also affected by severe back, groin, and leg pains [1]. Vascular-related complications are most commonly associated with the use of traditional compression haemostasis after coronary procedures (incidence, approximately 2–10 %). Such complications include hematoma formation, followed by bleeding, pseudoaneurysm formation, arteriovenous fistula formation, peripheral arterial thrombosis or embolism, and infections [2–4].

By the early 1990s, vascular closure devices were already in use for haemostasis after catheter removal. The devices were used as alternatives to traditional compression haemostasis. Among the various closure devices, the angioseal is notable for its ease of placement and is commonly used for haemostasis. The haemostatic effect of angioseal is achieved within 5.8–7.7 min, and patients only have to remain bedridden for 0.5–4 h after the procedure. Furthermore, the incidence of complications after angioseal haemostasis is 0.8–4.1 %. These factors allow for early return to routine activities [5–7]. The safety and efficacy of angioseal haemostasis after coronary procedures performed through femoral puncture have been confirmed [8, 9]. Angioseal allows for early return to routine activities; however, existing data on its effectiveness in decreasing complications is inconclusive. Some studies have reported that, compared with traditional compression haemostasis, angioseals achieved haemostasis faster, promoted early ambulation and early discharge, increased patient comfort levels, delayed bleeding complications, and resulted in a lower vascular complication rate [10–12]. However, a few studies have also shown an increased complication rate associated with the use of angioseals [13].

Factors other than haemostasis method have been reported to affect the complication rate. In particular, the complication rate was higher with the use of larger catheters [14] and varied with different access sites. The complication rates associated with radial, femoral, and brachial artery puncture sites were 0.0 %, 2.0 %, and 2.3 %, respectively [15]. Furthermore, the complication rates also varied with different coronary procedures, i.e., the rates were higher with interventional procedures (stent placement or angioplasty) than with diagnostic coronary procedures [2, 16, 17].

Patient-related factors influencing the complication rate include age, sex, medical history, body mass index (BMI), and blood pressure [4, 16, 18, 19]. Dumont et al. showed that certain factors such as female sex, age >70 years, presence of renal failure, and previous treatment with interventional coronary procedures were significantly related to vascular complications [18]. Another study focused on patients undergoing transfemoral coronary procedures and analyzed the odds ratios (ORs) for access site complications. The results showed increased complications among female patients aged >70 years and patients with renal failure, haemorrhagic diseases, chronic obstructive pulmonary disease, heart failure, and peripheral vascular diseases [4]. Berry et al. revealed a 1.6-times increase in the incidence of hematoma formation with every 5-unit increase in BMI [16]. In addition, the vascular complication rate was higher in patients with high systolic pressure during cardiac catheterization examination or catheter removal than in those with normal systolic pressure [18].

After a coronary procedure, the patients need to be confined to a bed for several hours. Back pain is a common complaint after coronary procedures and accounts for 35.8 % of all complaints. Back discomfort was reported to increase significantly in patients bedridden for more than 6 h [20]. Furthermore, the incidence of back discomfort was significantly lower in patients bedridden for less than 4 h than for those bedridden for more than 5 h; however, the vascular access site complication rate was not significantly different [21, 22]. Augustin et al. [23] assessed the incidence of back pain in patients with 3 or 10 h of bed confinement after percutaneous coronary intervention and found significant lower pain levels in the 3-h group. Taken together, the results suggest that patient comfort increases with a decrease in the duration of bed confinement [24]. However, Schiks et al. reported similar patient comfort levels after early and late ambulation for patients who underwent percutaneous coronary intervention [25].

Vascular closure devices such as angioseals were introduced to facilitate the reliable achievement of successful haemostasis at the access site and decrease discomfort caused by prolonged bed confinement. However, this product was only recently introduced in Taiwan, and its use is not currently covered by Taiwan’s National Health Insurance. Therefore, patients who select for angioseal haemostasis after coronary procedures need to pay an extra cost for this service. Consequently, the number of the studies in Taiwanese patients are limited. This study aimed to investigate the access site complication rate, predictive factors for complications, and patient comfort levels after coronary procedures with traditional compression or angioseal haemostasis.

## Methods

### Participants

This study adopted a purposive sampling method. The inclusion criteria were as follows: age, 20–80 years; the presence of coronary artery disease requiring transfemoral cardiac procedures (i.e. diagnostic or interventional angioplasty or stent placement) with a 6-Fr catheter; the presence of normal coagulation function before the procedure (platelet counts of 150,000–250,000/mm^3^); an international normalized ratio of <2 and the ability to walk independently without chronic lower back pain before the procedure. The procedures are elective and pre-scheduled. Those who received emergency/urgent diagnostic and interventional coronary procedures were not recruited in this study. The exclusion criteria were abnormal coagulation function, presence of severe liver disease and renal failure during dialysis. A sample size of 63 was estimated for each group based on calculation with a medium effect size of .5 and a power of .8 and from a two-tailed test with a significance level of α = .05 [26]. For comparison, the patients were divided into an angioseal group and a traditional compression group according to the patient’s choice of haemostasis method after the coronary procedure.

### Unit care protocol after diagnostic and interventional coronary procedures

Patient data were collected from a cardiac unit at a medical center in northern Taiwan. According to the unit protocol, the duration of bed confinement was different between diagnostic and interventional coronary procedures and between angioseal and traditional compression haemostasis methods. Furthermore, 6-Fr was the most commonly used catheter size for the diagnostic or interventional coronary procedures. In patients who underwent angioseal haemostasis after diagnostic catheterization, the catheter tube was removed immediately after the procedure and the angioseal was placed. A compression bandage was applied over the puncture site for 1 h. The patients remained in bed for another hour before they were allowed to leave their beds. If the procedure was interventional, a compression bandage was applied over the puncture site for 2 h. The patients remained in bed for another 2 h before they were allowed to leave their beds. In summary, this group remained bedridden for approximately 2–4 h.

In patients who underwent traditional compression haemostasis after diagnostic coronary procedures, the catheter tube was removed immediately after the procedure and a compression bandage was applied over the puncture site for 4 h. The patients remained in bed for another 2 h before they could raise the head of the bed up to 90°. Subsequently, the patients remained in bed for another 2 h before they were allowed to leave their beds. If the procedure was interventional, the sheath was removed after 4 h of bed confinement and a compression bandage was applied over the puncture site for another 4 h. The patients were bedridden for another 2 h before they could raise the head of the bed up to 90°. Subsequently, the patients remained in bed for another 2 h before they were allowed to leave their beds. In summary, this group remained bedridden for approximately 8–12 h.

In this center, heparin was the choice of anticoagulant, not bivalirudin or GPIIb/IIIA antagonists. We recorded the dose of heparin used during the coronary procedure.

### Data collection

Data collection and assessment were completed by a Master-prepared registered nurse (PJW). The research data were gathered between September 2011 and August 2012. The patients were identified according to the unit coronary procedure list. Informed consent was obtained before data collection. Complications were observed at different times according to the haemostasis method: after 12 h of bed confinement following catheter removal in the traditional compression group and at 1, 4, and 12 h after catheter removal in the angioseal group. To assess the level of soreness, numbness, and pain in the back, groin puncture sites, and legs in both groups, the numeric rating scale (NRS) was used 1 h after catheter removal and immediately before the patients left their beds. Demographic and disease-related information were gathered from medical record reviews.

The reliability of comfort level and complication assessment was ensured before data collection. The researcher (PJW), together with another experienced nurse, assessed 10 patients who underwent transfemoral coronary procedures to evaluate access site complications and patient comfort levels. Kappa statistics were used to assess inter-rater agreement with a coefficient of .9 (*p* = .03).

### Measures

Our questionnaire comprised the following three parts:

#### Demographic and disease-related Information

The demographic characteristics included age, sex, and BMI, while disease-related information included comorbidities, type of coronary procedure (diagnostic or interventional), duration of the procedure, anticoagulant dose, and number and location of stent placements.

#### Observation chart for puncture site complications

According to the study by Lee et al. [9], puncture site complications included bleeding (two 4 × 4 gauzes were totally immersed), oozing (the blood-tainted area was >3 × 3 cm^2^ on the gauze but did not reach the level of bleeding), haematoma formation (subcutaneous haematoma around the puncture site measuring ≥5 × 5 cm^2^ of its maximal diameter according to an inelastic ruler) and arteriovenous fistula formation (blood flow thrill sound confirmed by a stethoscope placed over the puncture site and by sonography performed by a cardiologist).

#### Recording chart for comfort levels

The numeric rating scale (NRS) was used to assess the degree of soreness, numbness, and pain in the back, legs, and groin puncture site. NRS is an ordinal scale with 0–10 ratings, where 0 indicates no pain and 10 indicates worst possible pain. The patients used this scale to express their current comfort levels. NRS is a common clinical tool with a similar measurement effectiveness, high test-retest stability, and a correlation coefficient of 0.80–0.96 [27–30].

### Statistical analysis

SPSS16.0 for Windows (Chicago, IL, USA) and SAS 13.3 (Cary, NC, USA) were used for data analyses. Descriptive statistics and inferential statistics, including t-test and chi-square statistics, were used to compare the outcomes between the two study groups. A logistic regression model was used to predict the important factors for access site complications. Furthermore, to compensate for the non-randomized design and few events of complications of this study, we used propensity score methods [31]. A propensity score was calculated and multivariate logistic regression analysis was performed and adjusted for the propensity score. All statistical tests used were two-tailed, with a significance level of α = .05.

### Ethics

This study was reviewed and approved by the Research Ethics Committee (No. 201108034RC) of National Taiwan University Hospital. The researcher explained the study purposes and procedures in detail and obtained written consent from each patient. The patients were free to withdraw during the study without any compromise in subsequent treatment.

## Results

**Fig. 1:**
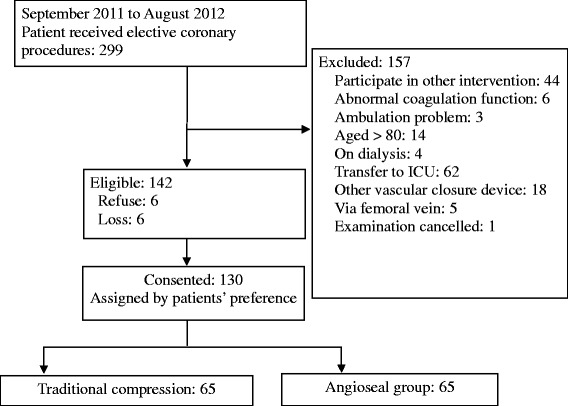
Flow chart for patient recruitment

From September 2011 to August 2012, 299 patients underwent elective coronary procedures. Among these, 142 met the inclusion criteria. Six rejected participation and six were lost to attrition. Totally, 130 patients were included (99 males, 76.2 %; mean age, 64.2 years; SD, 9.7 years; average BMI, 25.6; SD, 3.5; Fig. 1). There were no significant differences in demographic- and disease-related information between the traditional compression and angioseal groups (Table 1). During the study period, heparin was the only anticoagulant used for the coronary procedures. The average amount of heparin used for the patients was 4153.9 ± 3003.8 units.

**Table 1:** Demographic and clinical characteristics of the study sample

Variable	Total number (*n* = 130)		Traditional compression (*n* = 65)		Angioseal (*n* = 65)		Statistics	*p*
	n	%	n	%	n	%		
Gender							χ^2^ = 3.43	.06
Male	99	76.2	45	69.2	54	83.1		
Female	31	23.8	20	30.8	11	16.9		
Age M ± SD	64.2 ± 9.7		64.9 ± 9.3		63.4 ± 10.2		t = .86	.39
(range)	(32-80)		(42-79)		(32-80)			
BMI M ± SD	25.6 ± 3.5		25.5 ± 3.8		25.8 ± 3.2		t = -.56	.58
(range)	(17.3-34.4)		(17.6-34.0)		(17.3-34.4)			
Comorbidity								
Hypertension	92	70.8	48	73.9	44	67.7	χ^2^ = .34	.56
Hyperlipidemia	44	33.8	21	32.3	23	35.4	χ^2^ = .03	.85
Diabetic Mellitus	32	24.6	20	30.8	12	18.5	χ^2^ = 2.03	.15
Renal Disease	4	3.1	3	4.6	1	1.5		^#^.62
COPD	1	0.8	0	0.0	1	1.5		^#^.00
Total number of comorbidity M ± SD	1.3 ± 0.9		1.4 ± 0.9		1.3 ± 0.9		t = .37	.29
(range)	(0-4)		(0-4)		(0-3)			
Type of catheterization							χ^2^ = .56	.46
Diagnostic	43	33.1	19	29.2	24	36.9		
Interventional	87	66.9	46	70.8	41	63.1		
Time spent for catheterization (minutes) M ± SD	82.2 ± 42.1		82.2 ± 44.8		82.1 ± 39.5		t = .01	.99
(range)	(15-252)		(15-220)		(25-252)			
Dose of anticoagulant M ± SD	4153.9 ± 3003.8		4053.9 ± 3199.3		4253.9 ± 2816.1		t = -.38	.71
(range)	(0-12500)		(0-12000)		(0-12500)			
Number of stent placement							χ^2^ = 3.74	.15
0	58	44.6	34	52.3	24	36.9		
1	45	34.6	21	32.3	24	36.9		
2 and above	27	20.8	10	15.4	17	26.2		

*M* Mean, *SD* Standard deviation, *COPD* Chronic Obstructive Pulmonary Disease, *χ* ^*2*^ Chi-Square Tests, *t* independent t-test

^#^: Fisher’s Exact test

The complication rate was 3.8 % overall (*n* = 5), 0.0 % in the traditional compression group, and 7.7 % (*n* = 5) in the angioseal group. Three patients experienced oozing within the first hour after catheter removal (*n* = 3; 4.6 %), while two experienced oozing 4 h after catheter removal (*n* = 2; 3.1 %). The patient who exhibited oozing 4 h after catheter removal exhibited hematoma formation 12 h after catheter removal. There were no cases of bleeding or arteriovenous fistula formation. The chi-square with Fisher’s exact test revealed no statistically significant difference in the complication rate between groups (*p* = .06; Table 2).

**Table 2:** Comparison of access-site complication

Variable	Total number (*n* = 130)		Traditional Compression (*n* = 65)		Angioseal (*n* = 65)		Fisher’s exact test
	n	%	n	%	n	%	*p*
Complication							.06
No	125	96.2	65	100.0	60	92.3	
Yes	5	3.8	0	0.0	5	7.7	

A t-test was used to compare patient comfort levels between groups. With regard to back soreness 1 h after catheter removal, the average NRS scores were 2.4 ± 2.3 and 0.7 ± 1.9 for the traditional compression and angioseal groups, respectively (t = 4.53; *p* = .00). With regard to pain at the puncture site 1 h after catheter removal, the average NRS scores were 0.3 ± 0.9 and 0.0 ± 0.2 for the traditional compression and angioseal groups, respectively (t = 2.39; *p* = .02). With respect to back soreness while getting out of bed, the average NRS scores were 6.2 ± 2.6 and 2.5 ± 2.7 for the traditional compression and angioseal groups, respectively (t = 8.16; *p* = .00). Finally, with regard to leg numbness while getting out of bed, the average NRS scores were 0.5 ± 1.4 and 0.1 ± 0.5 for the traditional compression and angioseal groups, respectively (t = 2.32; *p* = .02).

In addition, comparison of average NRS scores for back soreness 1 h after catheter removal and while getting out of bed within groups showed an increase in score from 2.4 ± 2.3 to 6.2 ± 2.6 for the traditional compression group. Discomfort in this group changed from mild to moderate, and the difference reached statistical significance (paired t = -11.93; *p* = .00). The average NRS score in the angioseal group increased from 0.7 ± 1.9 to 2.5 ± 2.7, and although the change in discomfort levels from the first hour after catheter removal to the time of getting out of bed was statistically significant (paired t = -5.8; *p* = .00), discomfort was mild at both time points.

Univariate logistic regression analysis revealed five independent predictors of complications. These included age >70 years (OR, 10.29; *p* = .04), diabetes mellitus (OR, 1.39; *p* = .02), total number of comorbidities (OR, 2.73; *p* = .04), duration of the procedure (OR, 1.02; *p* = .03), and number of stents placed (OR, 3.05; *p* = .04). A propensity score was calculated with these significant covariates. Multivariate logistic regression analysis was performed and adjusted for the propensity score, which revealed that an age of >70 years was the only significant predictive factor, with an OR of 10.44 (95 % CI, 1.81–60.06; *p* = .009). This indicates that the incidence of complications was 10.44 times higher among patients aged >70 years than among those aged <70 years (Table 3).

**Table 3:** Factors associated with access-site complication

Variable	Complication		Uni-variate logistic regression		Multi-variate logistic regression	
	Yes	No	Odd ratio	*p*	Odd ratio	*p*
	n	n	(95% CI)		(95% CI)	
Gender				1.00		
Male	5	94	1			
Female	0	31	0.00(0.00 ~ --)			
Age				*.04		**.009
≦70 years	2	89	1		1	
>70 years	3	36	10.29(1.11 ~ 95.26)		10.44(1.81 ~ 60.06)	
BMI				.49		
≧24	3	82	1			
<24	2	43	0.46(0.05 ~ 4.25)			
DM				*.02		
No	2	96	1			
Yes	3	29	1.39(0.48 ~ 5.05)			
Type of procedures				1.00		
Interventional	5	82	1			
Diagnostic	0	43	0.00(0.00 ~ --)			
Method of haemostasis				1.00		
Angio-seal	5	60	1			
Traditional	0	65	0.00(0.00 ~ --)			
Dose of anticoagulant				.13		
>5001	1	18	1			
≦5000	4	107	0.24(0.04 ~ 1.52)			
Comorbidity			2.73(1.06 ~ 7.02)	*.04		
Time spend in catheterization			1.02(1.002 ~ 1.03)	*.03		
No. of stent placement			3.05(1.06 ~ 8.73)	*.04		

*BMI* body mass index

*p < .05; **p < .01

## Discussion

This study presented the following main findings: (1) the overall incidence of complications was not significantly different between the traditional compression and angioseal groups, (2) age >70 years was the only significant predictive factor for access site complications, and (3) comfort levels were higher in the angioseal group than in the traditional compression group.

There was no significant difference in the access site complication rate between the two groups. This finding is consistent with that of the study by Hashim et al. [32]. However, previous studies reported this rate to be 0.8–4.1 % for angioseal haemostasis [5, 6, 33]. A possible explanation for the higher incidence rate reported in the present study is the difference in observed complications and their operational definitions. These differences may have affected the research results. In previous studies, vascular complications included vascular injuries that required surgical repair, situations that required blood transfusions, hemorrhage with a haematocrit reduction of >15 %, infections leading to prolonged hospitalization, pseudoaneurysm formation, large (>6 cm) and small (<6 cm) haematoma formation, delayed haemorrhage, arteriovenous fistula formation, posterior abdominal cavity hemorrhage, and acute lower extremity ischemia. The definitions of complications in the previous studies are broader, and the complications are more serious. We only investigated four types of complications: oozing, bleeding, haematoma formation, and arteriovenous fistula formation. Five patients experienced oozing. Clinically, oozing is a mild vascular complication that can be resolved by reapplying pressure over the puncture site and prolonging the duration of bed confinement.

Some studies have reported significant differences in the access site complication rate between traditional compression and angioseal haemostasis. Arora et al. [11] and Chevalier et al. [12] reported higher complication rates with traditional compression than with angioseal haemostasis. However, Dangas et al. [13] reported opposite results that were inconsistent with ours. We summarized factors other than the different definitions of complications that may have affected our results. First, several vascular closure devices were used in the previous studies, and the sample size distribution was uneven across these devices; this affected the overall complication rate. Second, the patients were from multiple centers, and there was no consistency in the unit care protocol after coronary procedures among different hospitals; furthermore, the studies did not mention the timing of catheter removal, compression duration, and bed confinement duration. Third, the target patients belonged to a high-risk population that included patients aged >70 years, those with multiple cardiac catheterizations over the same site, those with a history of hypertension, those who used anticoagulants before catheterization, and those who required a larger catheter (8-Fr).

Our results showed that the incidence of oozing was 7.7 % (*n* = 5) in the angioseal group, and oozing occurred 1 h (*n* = 3; 4.6 %) and 4 h (*n* = 2; 3.1 %) after catheter removal. These findings were similar to the results of Cremonesi et al. [34], who reported that 78.3 % bleeding complications that did not require surgical treatment (bleeding or hematoma) occurred 4 h after examination. It is necessary to observe the puncture site closely at this critical timing for a bleeding condition. Our results are also similar to those of Botti et al. [35], who found that 4.2 % bleeding complications occurred 6 h after catheterization examination. These results indicate that close assessment of the puncture site 6 h after coronary procedures is helpful for observing oozing or bleeding.

Patient comfort levels were higher in the angioseal group than in the traditional compression group, as illustrated by back soreness and puncture-site pain 1 h after catheter removal and back soreness and leg numbness at the time of leaving the bed. These findings are consistent with our research hypothesis that patients with shorter durations of bed confinement have significantly lower discomfort levels. Our results are also similar to those of previous studies [1, 22, 23]. The average NRS score for back soreness at the time of leaving the bed was 6.2 ± 2.6 for the traditional compression group; this score was lower than the average score of 7.6 ± 1.5 for patients lying flat in bed, as reported by Chen and Wu [27]. The difference between our results and those of Chen and Wu can be attributed to the different timings of soreness assessment.

Previous reports have listed factors that can affect the complication rate after coronary procedures. These included catheter size, use of anticoagulants, choice of puncture site/haemostasis method, and, if intervention was executed, patient age, sex, medical history, BMI, and mean systolic blood pressure during examination and catheter removal processes [10, 11, 14]. We found higher ORs for patients aged >70 years, those with diabetes mellitus, and those with a greater number of stent placements. Specifically, the incidence of complications was 10.44 higher for patients aged >70 years than for those aged ≤70 years. This increased incidence was statistically significant and is similar to that reported by Piper et al. [4] and Dumont et al. [36].

### Limitations and suggestions

This study investigated the access site complication rate, predictive factors for these complications, and patient comfort levels after coronary procedures with traditional compression or angioseal haemostasis to provide new knowledge applicable to clinical care in Taiwan. However, this study had some limitations. First, the comparison was based on patient preference because of nonreimbursement for angioseal by Taiwan’s National Health Insurance. Nonrandomization and the small sample size limit generalization of the study results. Second, only angioseal was studied; other types of vascular closure devices were not included in the investigation. Therefore, the results cannot be extended to all patients in whom vascular closure devices are used. Third, only immediate complications could be observed, and there was no investigation into delayed hemorrhage or hematoma formation. These issues are worthy of further investigation.

## Conclusion

The overall complication rate was 3.8 % (*n* = 5) after transfemoral coronary procedures in this study. There was no statistically significant difference (*p* = .06) in the complication rate between the traditional compression and angioseal groups. Older age (>70 years) was the only independent significant predictor of access site complications. These findings suggest that the common access site complications after coronary procedures are relative minor. Although the use of angioseal after coronary procedures did not increase the incidence of complications, it increased patient comfort levels.
